# The Similarity between Species Composition of Vegetation and Soil Seed Bank of Grasslands in Inner Mongolia, China: Implications for the Asymmetric Response to Precipitation

**DOI:** 10.3390/plants10091890

**Published:** 2021-09-13

**Authors:** Yanyan Lv, Menghong Shen, Baoping Meng, Huifang Zhang, Yi Sun, Jianguo Zhang, Li Chang, Jingrong Li, Shuhua Yi

**Affiliations:** 1 Institute of Fragile Eco-Environment, Nantong University, Nantong 226007, China; lvyy18@ntu.edu.cn (Y.L.); zhf10658@ntu.edu.cn (H.Z.); sunyi@ntu.edu.cn (Y.S.); sezjg@ntu.edu.cn (J.Z.); 2 School of Geographic Science, Nantong University, Nantong 226007, China; 1822011031@stmail.ntu.edu.cn; 3 College of Geography and Environmental Engineering, Lanzhou City University, Lanzhou 730070, China; changli@lzcu.edu.cn; 4 Institute of Water Resources for Pastoral Area, Ministry of Water Resources, Hohhot 010020, China; lijinrong918@126.com

**Keywords:** asymmetry, vegetation, seed density, similarity index, unmanned aerial vehicle

## Abstract

The asymmetric response of productivity to precipitation was recently proposed as an early warning signal for the shifts in temperate grassland function in China. It was hypothesized that the asymmetry was influenced by the increased growth of the newly emerged seedlings from the soil seed bank. Therefore, the seed density in the soil seed bank and the similarity between species composition of the vegetation and the soil seed bank should be maximized where asymmetry was maximized. However, this knowledge was still limited and unconfirmed. In this study, the desert steppe, typical steppe and the transition zone between them (with the highest asymmetry) were selected for studying the similarity index in both 2018 (dry year) and 2019 (wet year). Plant species composition was monitored in situ using an unmanned aerial vehicle. Soil seed bank samples were collected, and the seed bank density and species composition were then examined and identified in the laboratory. Results showed that: (1) The variation in vegetation species richness between the two years was the highest (41%) in the transition zone (*p* < 0.05), while it was only 7% and 13% for the desert steppe and typical steppe, respectively. The presence of herbaceous species mainly caused the differences in variation among three grassland types. (2) Seed density was the highest in the transition zone (114 seeds/m^2^ and 68 seeds/m^2^ in the transient and persistent soil seed bank, respectively) (*p* < 0.05). Additionally, herbaceous species were the main components of the soil seed bank. (3) The similarity index was the highest in the transition zone (*p* < 0.05), with 38%/44% and 33%/44% for the transient/persistent soil seed bank in 2018 and 2019, respectively. Our study demonstrated that variation in vegetation species composition was very similar to the composition of the seeds accumulated in the soil seed bank. These results warrant further investigation for the mechanism of asymmetric response of productivity to precipitation.

## 1. Introduction

Grasslands play a crucial role in both carbon and water cycles. The semi-arid and arid grasslands are experiencing frequent and intense droughts [1,2], which inevitably cause changes in the function, structure, and composition of grassland ecosystems [3,4,5]. Sometimes, the changes are catastrophic and irreversible, particularly in the North American Great Plains [6]. Therefore, understanding the early warning signals has great importance for grassland ecosystem services and human well-being.

The early warning signals have received much attention in recent years [7,8]. The relationship between above-ground net primary production (ANPP) and precipitation was an essential predictive signal of grassland functions [9]. The nonlinear response of ANPP to precipitation (asymmetry) has either been studied with models [9,10] or tested in desert [11] and switchgrass [12]. Recently, Hu et al. (2018) found that the asymmetry was maximized at the transition zone between the desert steppe and typical steppe in Inner Mongolia, China [13], and it was proposed to be such a warning signal for the temperate grassland ecosystems [13]. These results suggested that in the extremely dry years the zone was more similar to the desert steppe, and in extremely wet years it was more similar to the typical steppe. Plant density variation [11] and drought tolerance of resident plants [14] have been posited to be the determining factors for the asymmetry. Studies also hypothesized that seedlings that emerged from the soil seed bank beneath bare ground strongly influenced the asymmetry [13,15,16]. However, empirical study of this is lacking.

The soil seed bank is defined as the viable seeds that exist on the soil surface or are buried in soil [17], which represents the memories of the last plant community and the development of a future plant community in the surrounding area [18]. It is generally considered that the soil seed bank is the seed resource of native vascular plants, and would supply most seedlings for vegetation regeneration [19,20]. It is well-known that the amount of newly emerged seedlings is not only related to soil seed bank density [21,22], but also seed germination status and species composition. The latter can be quantified as a similarity index between soil seed bank species and vegetation species composition [19,23]. There have been numerous studies that have investigated the density of soil seed banks or their similarity index in Inner Mongolia [24,25,26,27,28,29]. Seeds accumulated in the soil seed bank would rapidly complete their life cycle even with only a few tens of millimeters of precipitation in the transition zone [20]. However, previous studies were conducted with varying numbers and sizes of samples and collection times. Furthermore, few have studied different grassland types within different regions, which prevented our further understanding of the potential role of soil seed banks on the asymmetry. 

The main objective of this study is to provide evidence of the potential contribution to asymmetry with the measurement of the soil seed bank density and similarity index for three representative grassland types (desert steppe, typical steppe and the transition zone) across Inner Mongolia, China. Since the asymmetry was maximized at the transition zone, we hypothesize that the soil seed bank density and/or the similarity index are also maximized at this zone.

## 2. Methods

### 2.1. Study Area

**Figure 1:**
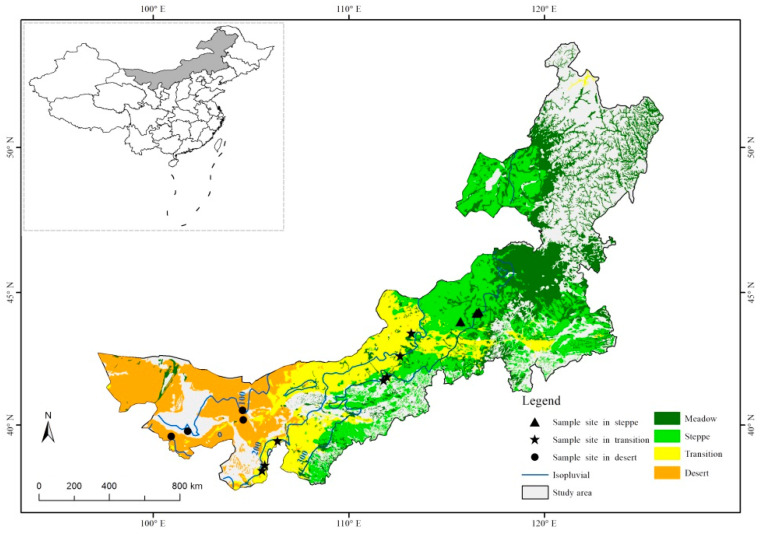
The study area of the desert steppe, transition zone, and typical steppe in Inner Mongolia, China. (Black points represent the sample sites where the vegetation composition was observed and soil seed bank samples were collected.).

This study was conducted in the desert steppe, typical steppe, and the transition zone across Inner Mongolia, China (Figure 1). The study area is distributed along a mean annual precipitation gradient ranging from about 100–300 mm, with approximately 65–70% of the total annual precipitation occurring in the peak growing season from June to August. The mean annual temperature ranges from 3–9 °C. Soils shift from calcic brown/desert steppe soils to chernozems and chestnut soils. Redundancy of plant functional types determines the stability of the grassland in Inner Mongolia [30]. Desert steppe is sensitive to species loss because of its limited functional redundancy, while typical steppe is less sensitive because its dominant grass genus, Stipa, is resistant to herbivory and drought [31]. Desert steppe is characterized by short (10–25 cm) xerophytic species (mainly short shrubs and semi-shrubs) with sparse cover (15–45%) and low species richness (5–10 species in 1 m^2^). The predominate plant species are *Reaumuria songarica*, *Potaninia mongolica*, *Sarcozygium xanthoxylon*, *Nitraria tangutorum* and *Kalidium foliatum*. The typical steppe is more productive than desert steppe and has continuous vegetation cover. Relatively xerophytic tufted perennial grasses mainly dominate the vegetation. The plant communities have a height of 14–35 cm, 30–50% cover and moderate levels of diversity (12–15 species in 1 m^2^) [32]. The predominating plant species are *Stipa capillata*, *Leymus chinensis*, *Artemisia capillaris*, *Artemisia scoparia* and *Chenopodium glaucum*. The vegetation of the transition zone consists of both shrubs and herbaceous species [33]. The species richness lies between the desert steppe and typical steppe. According to Hu et al. (2018) [13], the asymmetry was the highest in the transition zone, while lower in the desert steppe and typical steppe. This zone is more similar to the desert steppe in the extremely dry years, and it is more similar to the typical steppe in the extremely wet years. The sample sites were selected with relative uniformity and spatial representativeness of grassland types in the peak growing season. Because of the highest spatial variability within the transitional zone and the lowest spatial variability within the typical steppe zone, a total of 14 sample sites were designed in this study, i.e., 4 sample sites in the desert steppe, 7 sample sites for the transition zone, and 3 sample sites for the typical steppe to obtain vegetation information and soil seed bank sample collection (Figure 1).

The daily precipitation data in 2018 and 2019 were obtained from the nearest meteorological stations. The response of vegetation to precipitation had a certain delay for 50–60 days [34,35]. To measure the vegetation species variations with different precipitation between these two years in the peak growing season (July and August), the mean cumulative precipitation from May to June was gathered and is shown in Table 1.

### 2.2. Species Composition Observation by Unmanned Aerial Vehicle (UAV)

**Figure 2:**
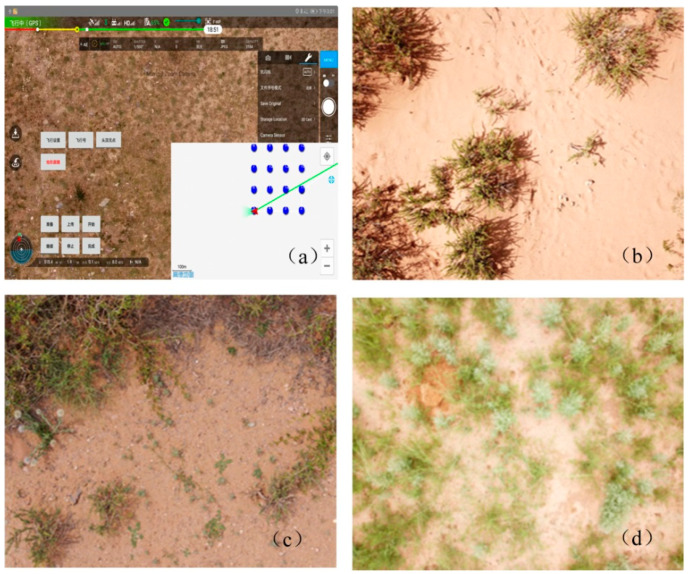
Unmanned aerial vehicle photographing (**a**); blue points represented the flight way points. Photos obtained from the desert steppe (**b**), where the predominate plant species is *Reaumuria songarica*; the transition zone (**c**), where the predominate plant species are *Reaumuria songarica*, *Astragalus membranaceus* and *Bassia dasyphylla*; and the typical steppe (**d**), where the predominate plant species are *Artemisia scoparia* and *Stipa capillata* in 2019, respectively.

The species composition of vegetation was observed based on a DJI drone (MAVIC Pro, DJI Innovation Company, China; with 3000 × 4000 pixels) in the peak growing season, from July to August in both 2018 and 2019. This observation time was consistent with the time for the ANPP dataset used in Hu et al. (2018) [13]. For each of the grassland types, BELT flight ways of the FragMAP system were used to take the aerial photos [36]. A BELT flight way consisted of 16 way points, which were evenly distributed within a 40 × 40 m plot (Figure 2a). The flight height was set to 2 m, and each photo (covering 2.6 × 3.5 m on the ground) was taken vertically at each way point (Figure 2). After automatic flying and aerial photographing, the drone was manually operated to take photos at a height of 0.5 m randomly in the plot. In each site, three BELT flight ways were set for replication. A total of 41 flight ways (14 sample sites) were set (Figure 1). The species composition was then identified visually and recorded all the species occurring within each aerial photograph [37,38]. Species richness is a simple and widely used index that indicates diversity of a study area [39]. In this study, we selected this index to assess the species composition within three grassland types. It was calculated as [40]:

N = number of species that appeared in each quadrant (mean value from 16 photographs).

Percentage of plant species variation (%) is used to express the changes in the species composition between the two years. The dry year, 2018, is considered as a reference and the absolute value of the % change to the wet year, 2019, was calculated.

### 2.3. Soil Seed Bank Sampling and Seed Density Test

**Figure 3:**
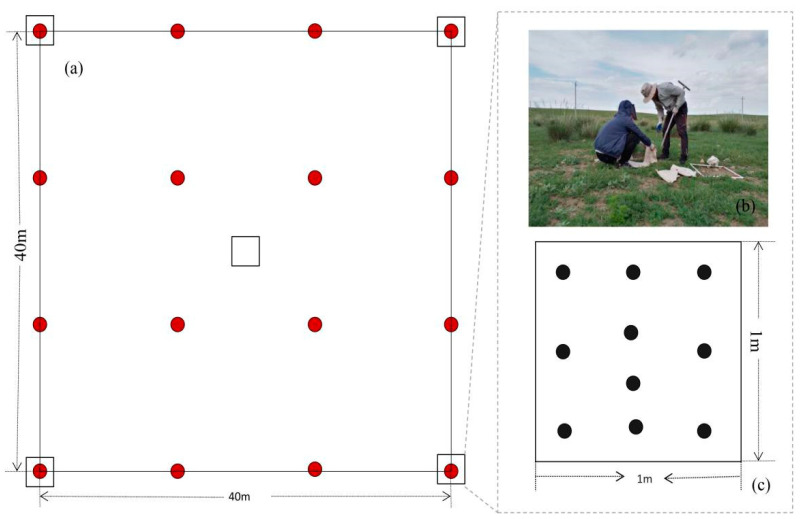
Diagram of soil seed bank sampling within the BELT plot (40 m × 40 m) (**a**); soil seed bank samples were collected from five quadrats (1 m × 1 m) (**b**,**c**). (Red dots denote the way points in the BELT flight way; the small black box represents the position of soil seed bank collection.).

The seed bank is characterized by the number of seeds in the soil, and it is changed by the input and output of seeds, being classified by its permanence in the soil as transient or persistent [41,42]. The soil seed bank samples were both collected in April (here identified as the transient soil seed bank) and July or August (as the persistent soil seed bank) [43]. In each sample site, one BELT plot (40 × 40 m) where the vegetation species composition was observed by the UAV was selected to collect a soil seed bank sample from. Four corners and central points in the BELT were chosen as a quadrat (Figure 3). Altogether, 5 sets of soil samples were taken from the whole BELT plot. A soil drill with a diameter of 3.5 cm was used to collect soil samples from the upper (0–5 cm) layers. Ten drilled soil cores of soils were then mixed as a soil sample. A total of 10 soil samples in each plot were collected (5 samples each for the transient and persistent soil seed bank, respectively). In total, 140 soil seed bank samples were collected.

Soil seed bank samples were then dried naturally and taken back to the laboratory for seedling emergence and species identification. Samples were sieved with a 0.3 mm aperture to remove impurities such as stones and litter. A 30 × 30 cm flowerpot was used as a germination bed. A thickness of 10 cm sterile sand was loaded and soil seed samples were then spread evenly on the surface. The flower pots were then put in an incubator with a temperature of 15~25 °C, alternating light and darkness (12 h/12 h). Enough water was added to keep the soil moist. Emerged seedlings were counted daily and identified when characteristics of the seedlings were evident. Whole experiments were considered to be finished when no new seedlings emerged in 7 days. At the end of the experiment, if un-germinated seeds existed, their viability was tested using tetrazolium methods [25]. Finally, the number of species was counted, and the density of soil seed banks (per m^2^) was calculated. In this study, three whole months were taken for seeds to completely germinate.

### 2.4. Similarity Index between Soil Seed Bank and Vegetation Species

The similarity index is an important indicator to evaluate the relationship between soil seed bank and vegetation species [44]. The Sorensen similarity index was used [33,45], which was calculated as follows Equation (1):

SC = 2W/(A + B)(1)

where A represented the number of species in the soil seed bank, B was the number of species in vegetation and W was the number of common species both in the soil seed bank and vegetation.

### 2.5. Data Analysis

One-way analysis of variance (ANOVA) was performed to test for differences in plant species richness, percentage of plant species variation, soil seed bank density, and similarity index for three grassland types using SPSS 18.0 software.

## 3. Results

### 3.1. Differences in Species Composition Variation among Three Grassland Types

**Figure 4:**
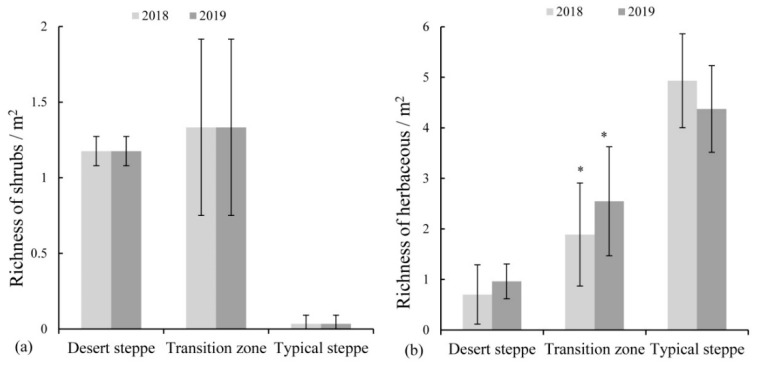
Species richness (species/m^2^) of shrubs (**a**) and herbaceous species (**b**) among three grassland types in 2018 and 2019. (* denotes a significant difference (*p* < 0.05) between two years.).

The floristic composition of the vegetation was clearly identified by aerial photos. In the desert steppe, shrubs mainly dominated the vegetation, including *Potaninia mongolica*, *Reaumuria songarica*, *Nitraria tangutorum* and *Sarcozygium xanthoxylon*, counting for about 75% of the total species (Table S1). There were no significant differences in shrub richness between 2018 and 2019 (*p* > 0.05; Figure 4a). The richness of herbaceous species increased in 2019, but was not significantly different from 2018 (Figure 4b). In the transition zone, shrubs’ richness showed no significant differences between the two years (Figure 4a). Nevertheless, the richness of herbaceous species significantly (*p* < 0.05) increased in 2019 (*p* < 0.05; Figure 4b and Table S2). In the typical steppe, species were dominated by herbs, including *Artemisia scoparia* and *Stipa capillata*; one shrub species (*Caragana microphylla*) was observed. The richness of herbaceous species showed no significant differences between 2018 and 2019 (*p* > 0.05; Figure 4 and Table S3).

**Figure 5:**
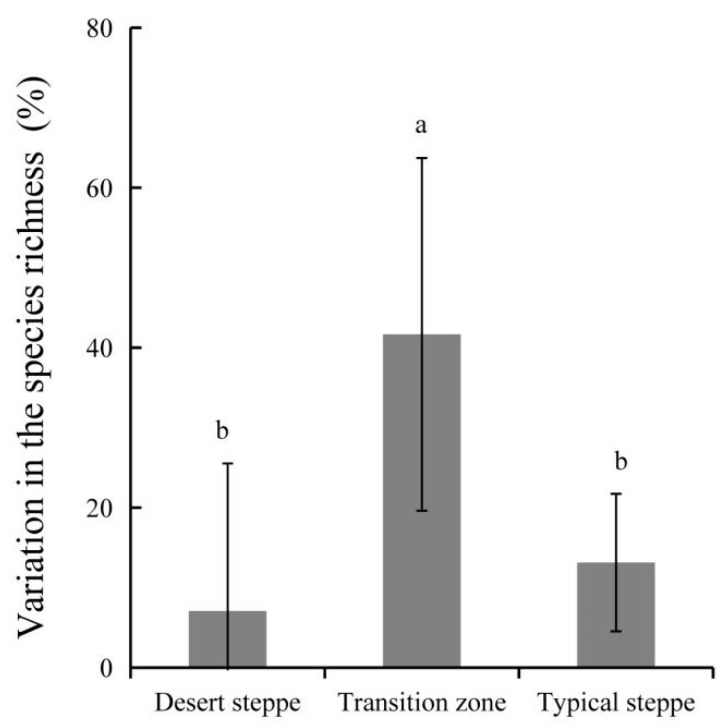
Variation (%) in the richness of plant species among desert steppe, transition zone and typical steppe. (Different superscripts denote significant difference (*p* < 0.05) in the variations of species among three grassland types of the desert steppe, transition zone and typical steppe.).

The variations in species richness between the two years differed among three grassland types; there were 41%, 7% and 13% variations in species richness in the transition zone, desert steppe and typical steppe, respectively (Figure 5). The variation of the transition zone was the highest (*p* < 0.05), while that in the desert steppe was the lowest. The highest variation in the transition zone was mainly caused by the increased number of herbaceous species (Figure 4).

### 3.2. Soil Seed Bank Density and Species Richness in the Soil Seed Bank

**Figure 6:**
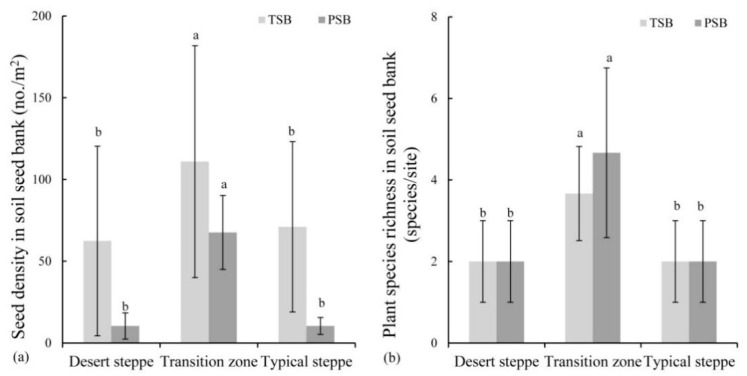
Density (**a**) and species richness (**b**) in the transient and persistent soil seed bank in the desert steppe, transition zone and typical steppe. (Different superscripts denote significant differences (*p* < 0.05) among three grassland types of desert steppe, transition zone and typical steppe.).

The transient soil seed bank density was higher than that of the persistent soil seed bank in each grassland type. For the transient soil seed bank, the seed density was the lowest in the desert steppe (62 seeds/m^2^), while highest in the transition zone (114 seeds/m^2^) (Figure 6a). All species present in the soil seed bank were identified as herbaceous species. *Cleistogenes squarrosa* was the most common species observed, and its seed numbers were the highest among the species in all three studied grasslands (Table S4). For the persistent soil seed bank, a similar pattern was observed (Figure 6a). Except for the shrub species of the Compositae family, all plant species were identified as herbaceous. Gramineae were the most common in the transition zone (Table S5).

For both the transient and persistent soil seed bank, herbaceous species were the dominant ones. Species richness in the soil seed bank in the transition zone (four and five species in the transient and persistent soil seed bank, respectively) was also significantly higher than the other two grassland types (*p* < 0.05) (Figure 6b).

### 3.3. Similarity Index between Soil Seed Bank and Vegetation

**Figure 7:**
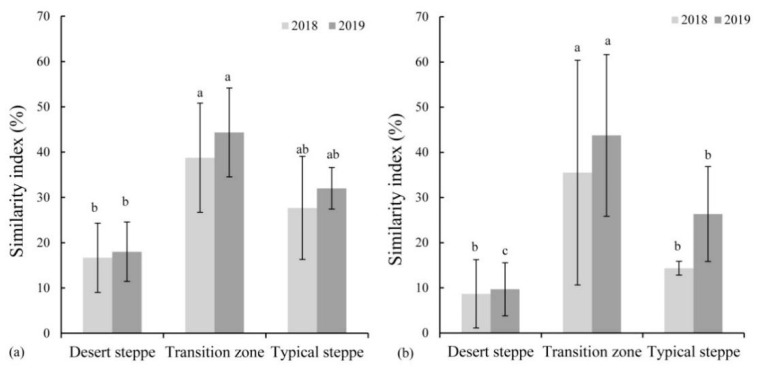
Similarity index (%) between the transient (**a**) and persistent (**b**) soil seed banks and species composition for the years 2018 and 2019 in desert steppe, transition zone and typical steppe. (Different superscripts denote significant differences (*p* < 0.05) among three grassland types of the desert steppe, transition zone and typical steppe.).

The similarity index between the transient soil seed bank and vegetation species composition in 2019 was higher than that in 2018 in all grassland types. The transient soil seed bank in the transition zone showed the highest similarity with the vegetation (38% and 44% respectively) in both years (Figure 7a), but it was not significant compared to the typical steppe (*p* > 0.05), while the similarity index was lowest in the desert steppe (*p* < 0.05). For the persistent soil seed bank, the similarity was higher in 2019, but not significant (*p* > 0.05) for each of the vegetation types (Figure 7b). The similarity index in the transition zone was the highest of all grassland types (*p* > 0.05).

## 4. Discussion

### 4.1. Highest Seed Density and Similarity Index in the Transition Zone

This study measured seed density and similarity index in the desert steppe, typical steppe and the transition zone between both. Results showed that the soil seed bank density and similarity index were the highest in the transition zone (Figure 6 and Figure 7). Several factors would explain this pattern: (1) Original plant assemblages [46] were the direct factor. The proportion of annuals and perennials differed in the soil seed bank and the vegetation. These differences possibly resulted in a low similarity between the soil seed bank and the vegetation. It was generally reported that perennials were found to be lacking in the soil seed bank, which meant less common species between the soil seed bank and the vegetation [47]. Thus, the exclusive species in the soil seed bank or vegetation increased the dissimilarity between the soil seed bank and the vegetation [48]. Besides, a different dispersal pattern leads to species differences between the soil seed bank and the vegetation. This was because the seeds were spread by the wind and spread far away from their mother plant. The low soil seed bank density was related to the scarcity of vegetation in the desert steppe [44]. The vegetation in the desert steppe was mostly composed of shrubs (Figure 4 and Table S1); however, we found no shrub seeds in the soil seed bank (Tables S4 and S5), consistent with He [33]. In the typical steppe, the vegetation was species-rich and dominated by herbaceous species (Table S3). Although these species produced plenty of seeds, most of them quickly emerged when falling in the soil and only a few seeds remained in the soil. In the transition zone, vegetation was composed of both shrubs and herbaceous species. (2) Abiotic factors such as wind regime, landform and soil condition [41,49,50,51,52] also affected soil seed bank density and similarity index. In our study area, the most important abiotic factors controlling both might be the soil moisture content. The water requirement for seed germination was different among species, e.g., for Gramineous (occurred in all study area) ones, the water requirement was low [53], but for species from the family Chenopodiaceae (occurred in the transition zone and typical steppe) (Tables S4 and S5), the requirement was higher [29]. When the precipitation could not meet the seed emergence requirement in the transition zone, seeds would be accumulated in the soil, resulting in a higher seed density. (3) Biotic factors, i.e., seed shape, seed size, the behavior of seed-eating animals, human disturbance, etc., were also main factors affecting soil seed bank density and similarity index [54,55,56,57,58,59]. Seed size was closely related to seed dispersal, and therefore seed density and similarity index. In the transition zone, the Chenopodiaceae, Compositae, Gramineae, Plantaginaceae, Umbelliferae were found. In contrast, only Compositae and Gramineae were found in the desert steppe and only Chenopodiaceae, Compositae, Gramineae and Liliaceae were found in the typical steppe (Tables S4 and S5). These species produced large quantities of seeds [60] and spread quickly [61,62].

### 4.2. Responses of Species to Precipitation and Implications

Asymmetry was maximized at the transition zone between the desert steppe and typical steppe in Inner Mongolia, China [13]. Hu et al. [13] hypothesized that the new seedlings that emerged from the soil seed bank beneath bare ground strongly influenced the asymmetry [13,15,16]. In this study, vegetation species richness was also maximized at the transition zone between dry and wet years (Figure 5). These species are mainly herbaceous species, including *Convolvulus ammannii*, *Erodium stephanianum*, *Astragalus membranaceus*, *Artemisia sieversiana* and *Bassia dasyphylla* in the wet year, and *Chenopodium glaucum*, *Artemisia scoparia*, *Cleistogenes squarrosa* and *Poa annua* in both the dry and wet years (Table S2). Moreover, the number of seedlings were increased dramatically in the wet year (2019). Among these nine species, five species were found in the soil seed bank and were accumulated with high density (Tables S2, S4 and S5). The precipitation is the main limiting factor for plant growth and seedlings emergency in the transition zone [33,44,63]. When the precipitation met the requirement for germination [64], large numbers of seedlings emerged. The relatively favorable environment and soil conditions were beneficial to herbaceous species growth [65,66].

In the desert steppe, although six species were found in the soil seed bank (Tables S4 and S5), only *Cleistogenes squarrosa* observed more seedlings on the vegetation in the wet year (Table S1). Besides, the vegetation species were rare and the similarity index as well as the soil seed bank density were low, thus the productivity will not be changed effectively with the precipitation [14,67]. In the typical steppe, *Cirsium japonicum* and *Convolvulus ammannii* were found in 2019. There was no obviously increased number of seedlings in this grassland type (Table S3). Because the vegetation coverage and growth rate were high and there was a linear correlation between productivity and precipitation [67], seeds accumulated in the soil seed bank had less effect on the vegetation.

The soil seed bank would supply most seedlings for vegetation regeneration [19]. The feasibility of vegetation restoration using the soil seed bank is largely dependent on its seed density and species composition [19,23]. Therefore, the increased number of vegetation species and similarity in the transition zone in a wet year implied that seeds accumulated in the soil seed bank might contribute to the asymmetry. However, long-term repeated monitoring of vegetation species with different precipitation regimes is required to test the asymmetry hypothesis.

### 4.3. UAV Application for the Mechanism Study of ANPP Asymmetric Response to Precipitation

Our study demonstrated that the soil seed bank potentially contributed to the asymmetry in the transition zone. The asymmetry was calculated based on ANPP data derived from the normalized difference vegetation index (NDVI) [13]. At present, the NDVI was one of the most widely used indices in grassland ANPP estimation [13,68,69]. Therefore, to test the hypothesis [13] of whether new seedlings or the growth of the existing plants contribute to the asymmetry, the dynamic changes in vegetation species and their NDVI should be monitored.

Traditional monitoring methods of the dynamic changes in the vegetation composition were usually limited by time, labor and resources [70]. The UAV method in this study, when appropriately calibrated, may overcome these limitations. Previous studies also demonstrated that the diversity indices derived from UAV photos are comparable with those from traditional methods [37,38,71]. For each set of way points, multiple flights with a fixed height (2 m) could be executed at different times to conduct repeated monitoring [36]. In this way, long-term monitoring over large regions can be realized. In addition to traditional cameras, multispectral cameras mounted on UAVs have become popular recently, which can be applied to monitor NDVIs with high spatial resolution [72]. We have performed preliminary experiments to take repeated photos over fixed locations using a UAV with a multispectral camera. These photos include five bands, and the NDVI of the regional shrubs and herbaceous species can be estimated in the ArcMap software. The proportions (%) of shrubs/herbaceous NDVI changes to the regional NDVI changes were then calculated. The preliminary results showed that the NDVI increased 20% under wet conditions in the transition zone. Although woody species contribute a large amount to the NDVI, their contribution to the NDVI changes with the precipitation is low (by 10%), while herbaceous species contribute 90%. In our future studies, we will monitor changes in species and their NDVI over large regions, including the desert steppe, typical steppe and the transition zone, to test the hypothesis of the asymmetric response of ANPP to precipitation using a UAV with a multispectral camera.

## 5. Conclusions

This study investigated the seed density and similarity index between vegetation and species in the soil seed bank in the desert steppe, typical steppe and transition zone. The results showed that both the seed density and similarity index reached the highest values in the transition zone, where the asymmetry was maximized. These results implied that new seedlings that emerged from the soil seed bank beneath bare ground might be the most important reason for the asymmetry. However, the dynamic changes in species and their NDVI should be monitored to test whether new seedlings or the existing plants contribute to the asymmetry most. A UAV mounted with a multispectral camera can be a feasible tool. The results would be helpful for vegetation restoration and desertification control in semi-arid regions of Northern China, as well as similar regions all over the world.

## Figures and Tables

**Table 1:** Cumulative average precipitation (mm) from May to June (data from meteorological stations) in desert steppe, transition zone and typical steppe in both 2018 and 2019.

Grassland Types	Cumulative Average Precipitation (mm) from May to June		Multi-Year Average Precipitation from May to June (mm)	Multi-Year Average Precipitation (~mm)
	2018	2019		
Desert steppe	10.53	34.43	21.15	115
Transition zone	32.25	73.25	53.40	200
Typical steppe	42.25	73.75	76.06	300

Note: The data of multi-year average precipitation from May to June (~mm) and multi-year average precipitation from 2000–2018 were obtained from https://www.worldclim.org/ (accessed on 20 November 2018).

## Data Availability

The data of multi-year average precipitation from May to June (~mm) and multi-year average precipitation from 2000-2018 were obtained from https://www.worldclim.org/ (accessed on 20 November 2018).

## Supplementary Materials

The following are available online at https://www.mdpi.com/article/10.3390/plants10091890/s1. Table S1: Vegetation species obtained by UAV in the desert steppe. Table S2: Vegetation species obtained by UAV in the transition zone. Table S3: Vegetation species obtained by UAV in the typical steppe. Table S4: Species identified in the transient soil seed bank for the desert steppe, transition zone and typical steppe. Table S5: Species identified in the persistent soil seed bank for the desert steppe, transition zone and typical steppe.
