# Supplementary material for: The Similarity between Species Composition of Vegetation and Soil Seed Bank of Grasslands in Inner Mongolia, China: Implications for the Asymmetric Response to Precipitation
Source: Plants (Basel). 2021 Sep 13;10(9):1890. doi: 10.3390/plants10091890 (PMC8467124; doi:10.3390/plants10091890)
Supplement: Supplementary file 1 [file plants-10-01890-s001.zip › plants-1327245-supplementary.pdf]

Supplementary Materials:

Table S1. Vegetation species obtained by UAV in the desert steppe

Grassland Types	Family	Species	Life style
Desert steppe	Rosaceae	*Potaninia mongolica*	S; P
	Tamaricaceae	*Reaumuria songarica*	S; P
	Zygophyllaceae	*Nitraria tangutorum*	S; P
		Sarcozygium xanthoxylon	S; P
	Gramineae	***Cleistogenes squarrosa*********	H; P
		Eragrostis pilosa	H; A
		*Phragmites australis**	(H); P
		Stipa glareosa	H; P
	Iridaceae	*Iris tenuifolia*	H; P

S: shrubs; H: herbaceous; A: annuals; P: perennials

* species just observed in 2019 (the wet year)

** species both observed in the wet and dry years (2018), but more in the wet years.

The bold marked species were observed both in the vegetation and soil seed bank.

The non-marked species were observed both in the dry and wet years.

Table S2. Vegetation species obtained by UAV in the transition zone

Grassland Types	Family	Species	Life style
Transition zone	Chenopodiaceae	*Kalidium foliatum*	S; P
	Rosaceae	*Potaninia mongolica*	S; P
	Tamaricaceae	*Reaumuria songarica*	S; P
	Zygophyllaceae	*Sarcozygium xanthoxylon*	S; P
		Nitraria tangutorum	S; P
	Chenopodiaceae	***Bassia dasyphylla****	H; A
		Chenopodium glaucum**	H; A
	Compositae	***Artemisia capillaris***	H; P
		Artemisia scoparia**	H; P(A)
		Artemisia sieversiana*	H; A
		Heteropappus altaicus	H; P
	Convolvulaceae	*Convolvulus ammannii**	H; P
	Geraniaceae	*Erodium stephanianum**	H; P
	Gramineae	*Agropyron cristatum*	H; P
		Cleistogenes caespitosa	H; P
		Cleistogenes songorica	H; P
		Cleistogenes squarrosa**	H; P
		Eragrostis pilosa	H; A

		Leymus secalinus	H; P
		Poa annua**	H; P
		Setaria viridis	H; A
		Stipa bungeana	H; P
		Stipa capillata	H; P
		Stipa glareosa	H; P
	Leguminosae	*Astragalus membranaceus**	H; P
	Liliaceae	*Allium mongolicum***	H; P

S: shrubs; H: herbaceous; A: annuals; P: perennials

* species just observed in 2019 (the wet year)

** species both observed in the wet and dry years (2018), but more in the wet years.

The bold marked species were observed both in the vegetation and soil seed bank.

The non-marked species were observed both in the dry and wet years.

Table S3. Vegetation species obtained by UAV in the typical steppe

Grassland Types	Family	Species	Life style
Typical steppe	Leguminosae	*Caragana microphylla*	S; P
	Chenopodiaceae	***Chenopodium glaucum***	H; A
	Compositae	***Artemisia capillaris***	H; P
		Artemisia scoparia	H; P(A)
		Artemisia sieversiana	H; A
		*Cirsium japonicum**	H; P
		Heteropappus altaicus	H; P
	Convolvulaceae	*Convolvulus ammannii**	H; P
	Gramineae	*Achnatherum splendens*	H; P
		Agropyron cristatum	H; P
		Cleistogenes squarrosa	H; P
		Leymus chinensis	H; P
		Phragmites australis	H; P
		Poa annua	H; P
		Setaria viridis	H; A
		Stipa bungeana	H; P
		Stipa capillata	H; P
	Leguminosae	*Astragalus membranaceus*	H; P
		Vicia sepium	H; A
	Liliaceae	***Allium mongolicum***	H; P

S: shrubs; H: herbaceous; A: annuals; P: perennials

* species just observed in 2019 (the wet year)

** species both observed in the wet and dry years (2018), but more in the wet years.

The bold marked species were observed both in the vegetation and soil seed bank.
The non-marked species were observed both in the dry and wet years.

Table S4. Species identified in the transient soil seed bank for the desert steppe, transition zone and typical steppe

Grassland types	Family	Species	Life style	Mean number of Seeds/m^2
Desert Steppe	Compositae	*Artemisia capillaris*	H; P	5
	Gramineae	***Cleistogenes squarrosa***	H; P	57
Transition Zone	Chenopodiaceae	***Bassia dasyphylla***	H; A	6
		Chenopodium glaucum	H; A	13
	Composita	***Artemisia capillaris***	H; P	2
		Artemisia scoparia c	H; P(A)	13
		Artemisia sieversiana	H; A	2
	Gramineae	***Cleistogenes squarrosa c***	H; P	65
		Poa annua c	H; P	1
		Stipa capillata	H; P	5
	Plantaginaceae	*Plantago minuta*	H; A(P)	5
	Umbelliferae	*Sphallerocarpus gracilis*	H; P	1
Typical Steppe	Chenopodiaceae	*Bassia dasyphylla*	H; A	5
		Chenopodium glaucum	H; A	4
	Compositae	***Artemisia scoparia***	H; P(A)	8
	Gramineae	***Cleistogenes squarrosa***	H; P	40
		Stipa capillata	H; P	11
	Liliaceae	***Allium mongolicum***	H; P	1

H: herbaceous; A: annuals; P: perennials
The bold marked species were observed both in the vegetation and soil seed bank.

Table S5. Species identified in the persistent soil seed bank for the desert steppe, transition zone and typical steppe

Grassland types	Family	Species	Life style	Mean number of Seeds/m^2
Desert Steppe	Compositae	*Artemisia sphaerocephala*	H; P	1
	Gramineae	***Cleistogenes squarrosa***	H; P	1
		Leymus secalinus	H; P	1
		Poa annua	H; P	8
Transition Zone	Chenopodiaceae	*Bassia dasyphylla c*	H; A	3
		Chenopodium glaucum c	H; A	1
	Compositae	***Artemisia sphaerocephala***	H; P	17
	Gramineae	*Cleistogenes squarrosa*	H; P	1
		Leymus chinensis	H; P	4
		Poa annua	H; P	32
		Stipa capillata	H; P	9
	Polygonaceae	***Polygonum sibiricum***	H; P	3
Typical Steppe	Chenopodiaceae	***Chenopodium glaucum***	H; A	1
	Compositae	***Artemisia capillaris***	H; P	2
		Cephalanoplos setosum	H; P	1
	Gramineae	***Cleistogenes squarrosa***	H; P	1
		Leymus chinensis	H; P	2
		Poa annua	H; P	1
		Stipa capillata	H; P	2
	Polygonaceae	*Polygonum sibiricum*	H; P	1

H: herbaceous; A: annuals; P: perennials
The bold marked species were observed both in the vegetation and soil seed bank.
